# Time to Emergence of the Lyme Disease Pathogen in Habitats of the Northeastern U.S.A.

**DOI:** 10.3390/insects16060631

**Published:** 2025-06-16

**Authors:** Dorothy Wallace, Michael Palace, Lucas Eli Price, Xun Shi

**Affiliations:** 1 Department of Mathematics, Dartmouth College, Hanover, NH 03755, USA; 2 Department of Earth Sciences, University of New Hampshire, Durham, NH 03824, USA; palace@guero.sr.unh.edu; 3 Department of Geography, Dartmouth College, Hanover, NH 03755, USA; lucas.e.price@dartmouth.edu (L.E.P.); xun.shi@dartmouth.edu (X.S.)

**Keywords:** Lyme disease, *Ixodes scapularis*, *Borrelia burgdorferi*

## Abstract

Ticks carry a range of pathogens, the best known of which causes Lyme disease, prevalent in the northeastern United States. Emerging diseases do not yet consist of a wide range of Lyme diseases, raising the question of how long it takes for a newly introduced tick-borne disease to establish itself. The aim of this study was to address this question, with the agent of Lyme disease used as the test case. A prior process-based model of the *Ixodes scapularis* (Say 1821) life cycle and the transmission of *Borrelia burgdorferi* (Burgdorfer 1982) between this tick and its various hosts was used to predict the dynamics of disease introduction into a new area. The importance of temperature, infection probabilities, and tick host populations, relative to that of other factors, was established by a global sensitivity analysis using Latin hypercube sampling. The results of those samples were analyzed to determine the time to near-equilibrium. Eight locations in New Hampshire were chosen for high/low temperature, high/low mouse, and high/low deer values. Mammal abundance was estimated by relating the known mammal density from previous studies to a MaxEnt analysis output. The time required to reach *Borrelia* endemicity in the ticks of New Hampshire ranged from 8 to 20 years in regions where the tick population is viable, with a strong dependency on susceptible tick host populations.

## Simple Summary

Lyme disease is contracted by humans through the bite of a black-legged tick. The pathogen is maintained in tick populations through reservoirs of tick hosts in the wild, including pathogen hosts such as the white-footed mouse and mobile tick hosts such as the white-tailed deer. The pathogen may be introduced into a new location by the arrival of infected ticks, but it may take years to establish at endemic levels. A process-based model describes the tick life cycle, host interaction, and disease transmission. A statistical model predicts the populations of deer and mice across New Hampshire. We combine both types of models to estimate how long it takes this disease to establish in the environment and to identify major contributing factors.

## 1. Introduction

Lyme disease in humans was first identified in the northeastern U.S. in the 1970s due to a cluster of cases in Lyme, Connecticut. Its agent, the bacteria *Borrelia burgdorferi* (Burgdorfer 1982) [1], and its vector, the tick *Ixodes scapularis* (Say 1821) [2], were identified in 1981 [3]. From 1985 to 2013, reported cases spread northward, with few initially reported in New Hampshire [4,5,6]. Lyme disease is now the most common arthropod-borne disease in the U.S. [3]. New Hampshire has one of the highest rates of Lyme disease in the United States, with it having been identified in all 10 counties, according to the New Hampshire Department of Health and Human Services. The ticks themselves are present throughout New Hampshire but are more prevalent in southern areas, according to recent passive surveillance [7].

The means of transmission is between ticks and susceptible reservoir hosts [8]. The geographic expansion of disease risk is likely due to tick hosts that are mobile, such as deer and ground-nesting birds [8,9]. Deer do not host the bacterial agent but may carry ticks that are already infected [8].

The vector *I. scapularis*, or the black-legged tick, is a hard-bodied tick that requires up to three blood meals to complete its life cycle. Four stages (egg, larva, nymph, and adult) are punctuated by blood meals obtained by questing behaviors that expose the tick to weather. The ability of the tick to mature to the next stage depends on its success in finding a host, which, in turn, depends on the abundance of and access to hosts [10,11]. Intermediate periods involve digestion and molting, which take place underground or in leaf litter [8,12]. The length of these intermediate periods depends on the temperature. Humidity is also a factor [13]. Therefore, the progression of Lyme disease across the landscape depends on the underlying ecosystem supporting the tick population.

A process-based model was used to simulate tick and host populations and disease transmission over time. A sensitivity analysis was conducted to confirm the importance of temperature and host distribution to tick populations and infection prevalence in ticks. A data-driven statistical model was used to estimate the abundance of two important tick hosts in New Hampshire. Eight locations were selected for further study and comparison using the process-based model.

This study shows the importance of temperature in determining the viability of tick populations and the importance of host distribution in determining the time to steady state of the percent of nymphal ticks infected, at which point the disease is completely endemic in the tick population. In addition, it is shown that the time to the endemicity of disease in the tick population ranges from 5 to 20 years, with a high dependence on the population of mice, which are competent *Borrelia* hosts, and a low dependence on the population of deer, which are not *Borrelia*-competent hosts but play a large role in feeding and transporting ticks to new locations.

## 2. Materials and Methods

This study adapted a prior model, which is described here, with the equations given in the Appendix A. This model was based on the life cycle, temperature dependencies, and host requirements of *I. scapularis*, tuned to the temperature profile of Hanover, NH, and adjusted with the day length-related diapause to match the qualitative characteristics observed in previous studies [13,14,15]. The model was the basis of a global sensitivity analysis, which established the importance of several parameters, including some involving tick hosts.

Two of the main tick hosts are mice and deer. A statistical model of the relative abundance of these hosts was developed for the state of New Hampshire. Many researchers have examined habitat use/selection in accordance with niche quantification by measuring the environmental parameters of habitat types [16,17,18,19,20,21,22]. The determination of factors and the relationship of multiple variables is appropriate for habitat use studies [23]. The studied habitat attributes of an organism are associated with the “realized” niche, since observational field data merely examine a possible niche volume and not the entire range that a species may exist in. An understanding of the habitat can greatly aid conservation efforts and ecological studies [24,25].

Some robust analytical methods use presence-only data, sampling from background data, to develop a statistical model that extrapolates across the landscape habitat probability [26]. One such method is maximum entropy modeling. Using MaxEnt software version 3.4.1 [27], we estimated a site probability map across the landscape. Researchers have used this habitat estimation method on regional to landscape scales to infer archaeological settlement patterns in the Brazilian Amazon (black earths) [28], Bolivian bamboo forests (geoglyphs) [29], and Michigan [30]. This modeling approach has also been used in Southern Maine to estimate the habitat probability of New England Cottontails [31].

Based on this analysis, eight locations were chosen for further analysis that vary by the temperature profile, habitat probability of susceptible rodent hosts, and habitat probability of disease-resistant deer hosts.

### 2.1. Model Overview

The process-based model used in this study was based on prior models that include the life cycle and disease dynamics of both the arthropod vector *I. scapularis* and its hosts [14,15,32]. This tick has a multi-stage life cycle from egg to adult, with three molts, three questing periods, and three blood meals. Temperature-dependent maturation rates control parts of the tick life cycle occurring off-host, while the availability of hosts determines on-host survival rates [13]. In addition, ticks have inactive periods in which they do not quest for a host; these appear to be unrelated to temperature and may be related to day length [33]. These periods are sometimes referred to as “diapause” and have also been incorporated into prior models to explain observed questing patterns in the field.

Six classes of host were included, categorized as competent (able to carry and transmit *B. burgdorferi*) or incompetent and mobile or stationary. Competent hosts were further sorted into infectious or susceptible hosts. Competent stationary hosts are small rodents, in particular, the white-footed mouse (*Peromyscus leucopus* (Thomas 1895) [34]), which is the primary host of larval ticks. Competent mobile hosts are ground-nesting birds. Incompetent stationary hosts include mid-size animals, such as opossum. Incompetent mobile hosts are exclusively white-tailed deer (*Odocoileus virginianus* (Zimmerman 1780) [35]), which, while unable to transmit disease, can transport an infected nymph for a considerable distance. Each of these hosts has simple population dynamics limited by an environmental carrying capacity, as well as a per-individual capacity for hosting ticks [11]. Equations and default model parameters are given in Appendix A.1. All parameters are based on prior work with this model, in which multiple sources of data in the northeast were used either to estimate a parameter directly or to adjust parameters to produce outputs comparable to those observed in field studies [14].

### 2.2. Sensitivity Analysis

The model for the tick population and disease dynamics has 71 parameters describing temperature effects, host dynamics, diapause due to day length, the efficiency of questing for hosts, and the carrying capacity for both hosts and ticks on hosts. A global sensitivity analysis was performed using MatLab’s Latin hypercube sampling code, with all 71 parameters, repeated 100 times [36]. The specified range for each parameter was ±20% of the default parameters, as shown in Table A1 in Appendix A.2. The initial conditions were drawn from prior work.

The model describes 1 km^2^ in which ticks are already present but not yet infected. The initial conditions were set for 1 January of year 1, with 6,453,100 eggs, 4,856,100 engorged uninfected larvae, and 291,360 engorged uninfected nymphs. Disease was introduced by assuming that 100 infected nymphs were present on a deer. The remaining tick categories were all set to zero. The number of eggs, percent of nymphs infected, and percent of adults infected on 1 January of year 46 were calculated for each of the 100 runs, and the correlation coefficient was obtained for each of the 71 variables calculated.

Although there was a large annual variation in populations, the value of these populations on 1 January reached equilibrium, as did the infection prevalence in nymphs and adults. The approximate time to 1 January equilibrium was calculated as the first year in which the values were within a given threshold of the final year’s value, for each of the 100 runs specified in a hypercube sample. As the number of eggs was large, the threshold for eggs was set to 50,000. The other two quantities are given as percentages, with a threshold of 0.02 or within 2% of the final value for the “percent infected” nymphs and adults. All measures were taken on Jan 1 of each year, as only a few stages are present at that point. Time series were graphed for the three quantities of interest for all 100 runs to visually confirm calculations.

### 2.3. MaxEnt for Host Distributions

Statistical models were used to determine the locations of high and low deer or mouse densities. Spatial modeling was performed using Maximum Entropy Species Distribution Modeling Software 3.3 [26]. Geospatially derived layers were used to calculate the driving factors for site location and to estimate the probability of sites being suitable for a given species across the research domain, in essence a habitat probability map. The use of maximum entropy modeling allowed us to characterize the driving environmental and spatial indices for multiple species. Species point information was used for two species of host vectors (white-footed mouse and white-tailed deer).

The source locations for deer and mouse were obtained from the Global Biodiversity Information Facility (GBIF), an international network and data infrastructure funded by the world’s governments and aimed at providing anyone, anywhere with open access to data about all types of life on Earth. For mice, 1704 location/occurrences in NH were used. One study was removed from the analysis that was very localized. For deer, 1418 location/occurrences in NH were used.

The MaxEnt model was run with cross-validations when appropriate. Iterations were run using a convergence threshold set to 0.0001, with a default prevalence of 0.5. Response curves were generated for each input variable in our MaxEnt model. Jackknife tests were run to examine the importance, or relative contribution, of each variable to the model. The jackknife tests allowed for an examination of how a given variable contributed to the overall model, both when it was used exclusively to build the model and when it was excluded from the model. The jackknife tests were performed for both training and test datasets. The area under the curve (AUC) statistic was used to gauge the predictive capacity of the model and how well it performed compared with a null model. Models with AUC values that are greater than 0.75 are considered to predict the test point distribution accurately [30].

Geospatial layers provide insights into the distribution and driving attributes of ecological phenomena, such as species distribution. A wide range of variables that were likely to be insightful and exploratory were utilized. The following layers are described below, and their inclusion in specific model ensemble runs are mentioned in each of the specific sections. Table A2 describes each dataset and its spatial resolution. Table A3 and Table A4 give estimates of the relative contributions of the environmental variables to the MaxEnt model for *P. leucopus* and *O. virginianus,* respectively. The MaxEnt model was run at a 1 m resolution across the entire state of New Hampshire. Imagery or geospatial datasets that had a lower resolution were resampled to 1 m for modeling purposes.

### 2.4. Specific Locations

Based on the results of the MaxEnt model and these measurements, eight locations were chosen in New Hampshire with either high or low temperature profiles, high or low *P. leucopus* densities, and high or low *O. virginianus* densities. The MaxEnt model was used to find locations with high and low precipitation and temperature, as well as high and low probabilities of mouse and deer habitats. These locations were then used to extract the time series of meteorological time series data to drive the tick model. Temperature data were fit with a Fourier series to parameterize the model for each location. The temperature data were obtained from Daymet [37]. The 8 locations were not field-based or sampling locations. These locations were used to extract long-term meteorological data on temperature and precipitation to drive the model.

Recent population density estimates from around the state range from generally less than 4 deer per square mile of habitat in the White Mountains to 18–24 per square mile in more southern locations, and, as mentioned above, they have been averaging at about 12 per square mile statewide [38]. For low deer populations, 1.54 deer per km^2^ (4 per square mile) was used, and for high deer populations, 7.72 per km^2^ (20 per square mile) was used.

Although multiple rodents and other small mammals may carry *B. burgdorferi*, the white-footed mouse is an important reservoir species for *P. leucopus* [8]. Donnelly et al. found population densities of 5 to 39 per hectare, or 500 to 3900 per km^2^ [39]. A separate study by Wolff et al. found that home ranges averaged at 590 m^2^, with a minimum size of 500 m^2^, consistent with Donnelly et al. [40].

## 3. Results

### 3.1. Model Performance

Tick populations have large seasonal effects, with questing adults present early and late in the season and questing nymphs found primarily in the middle of summer. As the Lyme disease pathogen advances in tick and host populations, little seasonal effect was seen in the model on the percent infected nymphs or adults. The final percent infected nymphs ranged widely among the 100 runs, from 6 to 20%, while the final percent infected adults ranged from 10 to 20%.

### 3.2. Sensitivity Analysis

**Figure 1:**
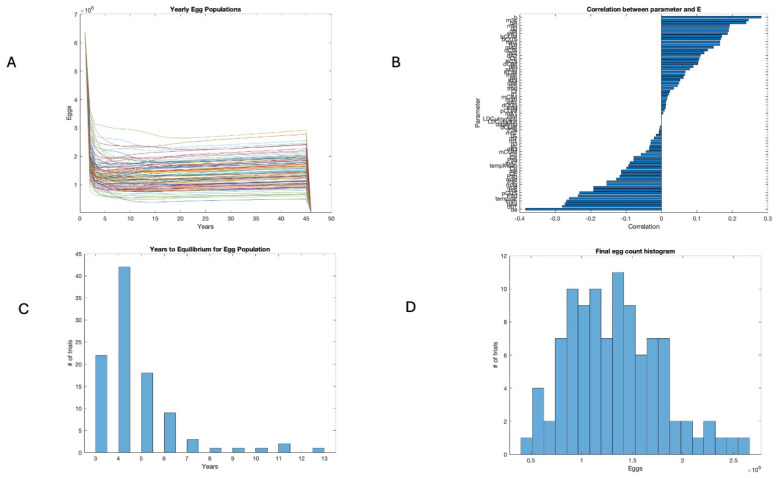
Egg population. (**A**) Time series for 100 runs with varied parameters. (**B**) Global sensitivity of egg population. (**C**) Years to equilibrium. (**D**) Final egg populations.

Egg populations were measured on 1 January of each year of the model to suppress seasonal effects. Equilibrium was reached in every run, which can be visually checked in Figure 1A. The final number of eggs was 0.5 × 10^6^ to 2 × 10^6^ across all runs, as shown in Figure 1D. The parameters with the strongest positive correlation with egg abundance were the birth rate (b), the transition of feeding larvae to engorged nymphs (m3c), and the probability that a questing adult finds any host (qA). Those with the strongest negative correlation with egg abundance were the death rate of eggs (de), death rate of engorged nymphs (dn1), and death rate of engorged adults (dA4). However, no single correlation was large, and many parameters had a similar effect size, as seen in Figure 1B. The time to egg equilibrium was fairly short, as seen in Figure 1C.

**Figure 2:**
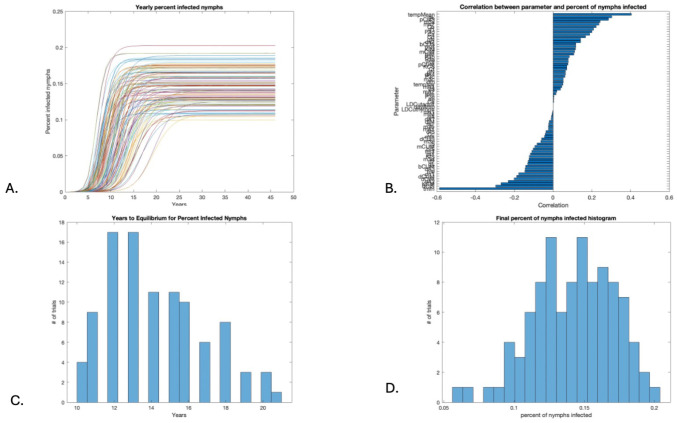
Percent infected nymphs. (**A**) Time series for 100 runs with varied parameters. (**B**) Global sensitivity of percent infected nymphs. (**C**) Years to equilibrium. (**D**) Final percent infected nymphs.

The percent infected nymphs was measured on 1 January and can be seen to reach equilibrium in Figure 2A. The parameters with the strongest positive correlation with the percent of nymphs infected at the end of the run were the mean annual temperature (tempMean), the probability of a feeding larva picking up infection from an infected host (pL), the probability of a competent uninfected stationary host (i.e., a rodent) picking up infection from an infected feeding tick (pCUS), the temperature dependence of the nymph maturation rate (tmfn), the death rate of competent uninfected stationary hosts (i.e., rodents) (dCIS), and the carrying capacity of competent mobile hosts (i.e., birds) (KCM). In this case, the parameters with the strongest correlation stood out a bit more than those for egg populations, as seen in Figure 2B. The range of times to equilibrium and the infection prevalence in nymphs at equilibrium are shown in Figure 2C and Figure 2D, respectively.

**Figure 3:**
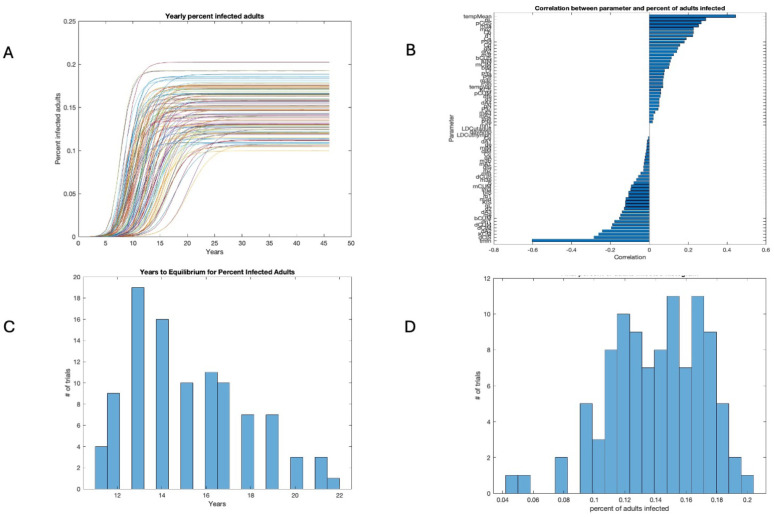
Percent infected adults. (**A**) Time series for 100 runs with varied parameters. (**B**) Global sensitivity of percent infected adults. (**C**) Years to equilibrium. (**D**) Final percent infected adults.

The percent infected adults was similar, with a year longer time frame due to the extra year of maturation, as seen in Figure 3A,C. The parameters with the strongest positive or negative correlation with the percent infected adults were the same as those for nymphs, as seen in Figure 3B. The final infection rates were a bit higher than those for nymphs, as seen in Figure 3D.

### 3.3. Years to Equilibrium and Endemicity of Disease

The initial conditions were the same for all runs, but the equilibrium values for the final egg populations varied with the parameter shifts in the 100 runs shown in Figure 1D. Figure 1C shows a histogram of the time to equilibrium, with the majority of runs approaching near-equilibrium in 4 years or less. In contrast, the number of years to equilibrium for the percent infected nymphs ranged from 8 to 21 years, as seen in Figure 2C, while the number of years to equilibrium for the percent infected adults was, predictably, a year longer, ranging from 10 to 22 years, as seen in Figure 3C. The sensitivity analysis suggests that temperature and host abundance strongly affect the time to steady state.

### 3.4. MaxEnt for Host Distributions

**Figure 4:**
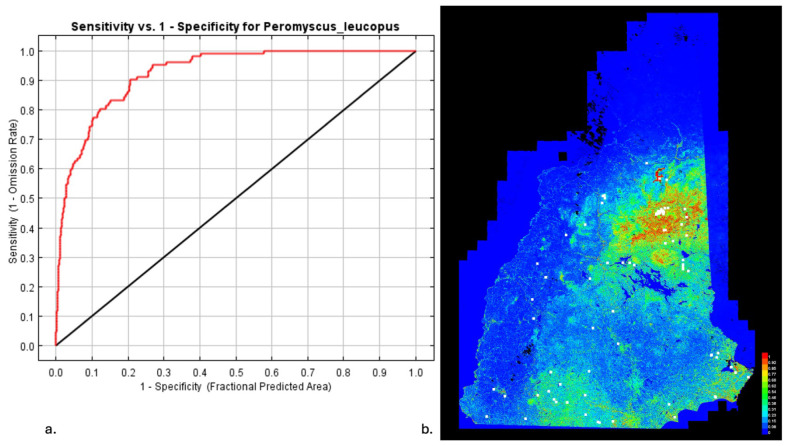
Density results for *P. leucopus*. (**a**) Sensitivity vs. specificity for the MaxEnt prediction of *P. leucopus* density. Red is the training data (AUC = 0.925), and black is the random prediction (AUC = 0.5). (**b**) MaxEnt results for *P. leucopus* density in the state of NH. Warmer colors show areas with better predicted conditions. White dots show the presence locations used for training, while violet dots show the test locations to be modeled.

**Figure 5:**
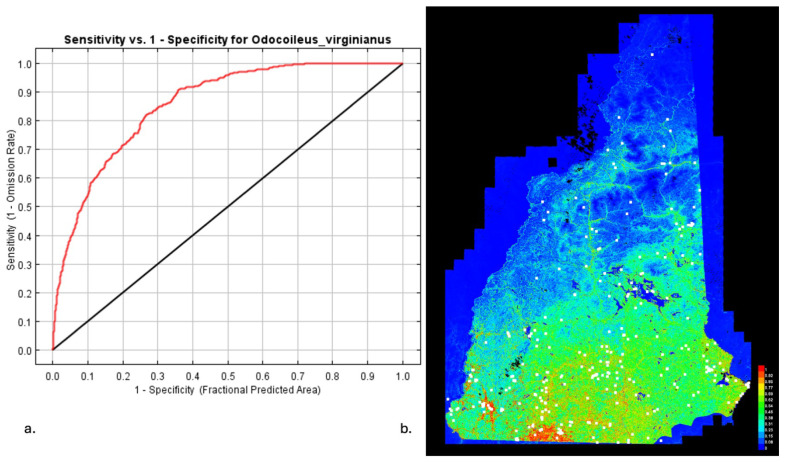
Density results for *O. virginianus*. (**a**) Sensitivity vs. specificity for the MaxEnt prediction of *O. virginianus* density. Red is the training data (AUC = 0.856), and black is the random prediction (AUC = 0.5). (**b**) MaxEnt results for *O. virginianus* in the state of NH. Warmer colors show areas with better predicted conditions. White dots show the presence locations used for training, while violet dots show the test locations to be modeled.

Figure 4 and Figure 5 show the results of the analysis of the mouse and deer distributions in New Hampshire. Figure 4a and Figure 5a show the sensitivity versus specificity for the mouse and deer predictions. In both cases, the AUC was over 0.75, indicating a good prediction. The MaxEnt model provides an indication of the key habitat of the modeled species, and this is often associated with a higher population density. Note that areas of high/low habitat probability are not particularly correlated between mouse and deer. This allows for a selection of sites with a high/low temperature, mouse habitat probability, and deer habitat probability.

### 3.5. Comparison of Eight Locations

**Figure 6:**
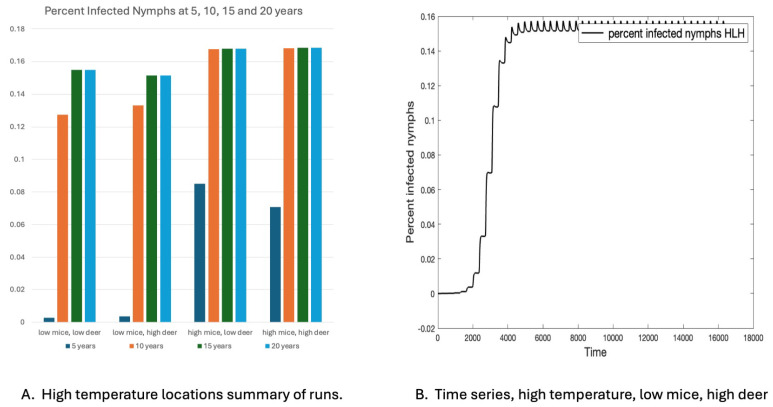
Percent infected nymphs in four high-temperature locations. (**A**) Percent infected at 5, 10, 10, 15, and 20 years. (**B**) Time series for a site with a warm temperature and low mouse and low deer populations.

The eight locations in New Hampshire chosen for comparison varied by temperature and host density. Densities were set to high and low values found in the literature. The four locations with low temperature profiles could not sustain the tick population. The four locations with warm temperature profiles were able to sustain tick populations. These are summarized in Figure 6. The time series for one of these is shown in Figure 6B, with seasonality. The sites with higher mouse populations reached a steady state earlier than those with low mouse populations, as shown in Figure 6A. Deer populations did not seem to make a large difference.

## 4. Discussion

This study makes use of a model previously described in the literature [14,15,32]. The maturation rates of *I. scapularis* in the model are temperature-dependent and parameterized based on experiments by Ogden et al. [13]. These dependencies prevent efficient maturation in regions with short warm seasons. The strong link between disease prevalence and temperature is confirmed in this study by the sensitivity analysis, which indicates that the mean annual temperature is one of the parameters to which the *B. burgdorferi* prevalence in nymphs was most sensitive, consistent with other studies [14,32,41,42,43,44]. In the four specific locations modeled that had a low mean annual temperature, the model showed tick populations declining to zero over time. Two caveats must be mentioned about this result. First, it may well be that some microclimates in a region may be quite cold, while others nearby could be warm enough to sustain tick populations. Therefore, it would not necessarily be true that there are no ticks in a given town or square kilometer of land. Second, ticks spend much of their maturation time hiding in soil or leaf litter, or even in animal dens, which have warmer temperatures than the air during the winter [8]. Measurements of these subsoil habitats would be useful in improving the temperature dependence of the model. It is worth noting, however, that passive tick surveys do not report ticks from colder regions of New Hampshire [7].

The strong links between Lyme disease and host populations are also confirmed by the sensitivity analysis of the model. Two of the most influential parameters were the death rate of competent uninfected stationary hosts (i.e., rodents, such as mice) and the environmental carrying capacity of *Borrelia*-competent mobile hosts (i.e., ground-nesting birds). The default parameters for the sensitivity analysis included the populations of these two categories, as well as two types of *Borrelia*-incompetent hosts. The difficulty of estimating animal populations across a region was addressed through MaxEnt software, which was able to estimate the relative abundance of the *Borrelia*-competent white-footed mouse and the *Borrelia*-incompetent deer populations across the state of New Hampshire. For the four warm-temperature sites, the rate of disease establishment at a given location had a strong dependency on mouse populations but no observable dependency on deer populations. This dependency only concerns the time to pathogen equilibrium and the prevalence of the pathogen in ticks. It does not reflect the number of ticks present, which certainly depends upon all hosts, nor does it represent the number of infected ticks, which determines the disease risk for humans.

Based on the sensitivity analysis, the range of time to disease endemicity in nymphs varied from 5 to 20 years, depending on the parameters, with 12 years being the mode of the distribution. This finding is consistent with the reported spread of Lyme disease in New Hampshire between 1998 and 2019, which increased dramatically in 20 years [5]. Because deers are highly mobile transporters of ticks, infected or not, it is likely that disease enters a town from a neighboring town. The time lag between disease reported in a county adjacent to one that has already had cases is estimated to be 7 years [45].

The analysis of the four warm-temperature sites showed the model to be at near-equilibrium in 10 years for high mouse populations and at equilibrium at 15 years for low mouse populations, consistent with the sensitivity analysis. As *Borrelia*-competent hosts, mice are believed to play a major role in the transmission of the Lyme disease spirochete [8,11,46,47]. Both the sensitivity analysis and the model results for warmer sites confirm this conclusion.

Sites with warmer temperatures were chosen in the towns of Plymouth (low mice and low deer populations), Madison (high mice and low deer populations), Deerfield (low mice and high deer populations), and Jackson (high mice and high deer populations). Jackson had 78.3 and 59.2 cases of Lyme disease per 100,000 in two five-year intervals (2005–2009 and 2010–2014). Madison had rates of 33.8 and 117.7 in the same intervals. Plymouth (14.3 and 62.4) and Deerfield (74.7 and 225.3) were the sites with lower mouse populations [48]. It is clear from these data that the eventual prevalence of disease in the human population is not particularly related to the time frame of disease establishment in the tick population. In other words, this study does not measure the risk of disease for humans. That risk is present long before the tick infection rate reaches a steady state, and it is dependent on many factors. These include human behaviors such as picking up the pathogen in one area and reporting the associated disease in another, the time spent outdoors, and other behaviors. Additionally, areas with low mice populations may offer an abundance of other disease reservoirs not included in this model.

## 5. Conclusions

In this study, we addressed the question of how long it takes a newly introduced disease to establish in a new location, using a process-based model of the tick life cycle and data-driven estimates of host populations. We found a time range from 5 to 20 years, depending on the tick and disease host populations and temperature. Model tick populations were not sustained at very low temperature ranges. In warmer temperature ranges, the disease prevalence in ticks was highly dependent on mouse (or other competent host) populations.

Although based on the transmission of *B. burgdorferi*, this model also has implications for other tick-borne pathogens, such as those responsible for anaplasmosis (*Anaplasma phagocytophilum*) and babesiosis (*Babesia microti*) [49]. Although currently less prevalent, one may expect these pathogens to also spread geographically. Based on the case of *B. burgdorferi*, this study may provide insights into the future spread of other pathogens by *I. scapularis*.

Most aspects of the model involve the life cycle, hosts, and temperature dependencies of *I. scapularis*, which is the same vector for other pathogens. The main difference in modeling other diseases transmitted by *I. scapularis* is the variation in the transmission rates between the tick and the host and the demographics of competent versus incompetent hosts for a different disease. The model for *B. burgdorferi* transmission is highly sensitive to the transmission rates between the vector and the host. This result indicates that measuring vector–host transmission rates for emerging tick-borne diseases should be a high priority.

## Data Availability

This project made use of MaxEnt software and data, Landsat 5 TM tiles https://earthexplorer.usgs.gov (accessed on 11 January 2024), Landsat 7 ETM+ tiles https://earthexplorer.usgs.gov (accessed on 11 January 2024), NLCD 2006 https://doi.org/10.5066/P9HBR9V3 (accessed on 11 January 2024), NLCD 2011 https://doi.org/10.5066/P97S2IID (accessed on 11 January 2024), TRMM 3B42 RT tiles https://disc.gsfc.nasa.gov/datasets/TRMM_3B42_V7/summary (accessed on 11 January 2024), NAIP 2009 https://www.fsa.usda.gov (accessed on 11 January 2024), NAIP 2011 https://www.fsa.usda.gov (accessed on 11 January 2024), BioClim (WorldClim), Daymet data https://daymet.ornl.gov (accessed on 11 January 2024), and public data from the New Hampshire Department Health & Human Services (DHHS) Data Portal https://wisdom.dhhs.nh.gov/wisdom/ (accessed on 7 March 2025). The source locations for deer and mouse were obtained from https://www.gbif.org/ (accessed on 11 January 2024).

## Appendix A

### Appendix A.1. Equations and Parameters

Tick eggs develop into hardening larvae, followed by a questing stage:

(A1) $$\begin{array}{cc} \dot{E} & =b*A_{4}-m_{Etemp}E-d_{e}E \end{array}$$

(A2) $$\begin{array}{cc} \dot{L_{1}} & =m_{Etemp}E-d_{1}L_{1}-m_{1}L_{1} \end{array}$$

(A3) $$\begin{array}{cc} \dot{L_{2}} & =m_{1}L_{1}-d_{2}L_{2}-m_{2}L_{2} \end{array}$$

In all relevant equations, the attachment process for successful feeding is described by an expression that is the product of the maturation rate, the prior population, a functional form *F* approaching zero as the total on-host carrying capacity is reached, and a ratio *Q* describing the probability of finding a host of a given type, *X*. Six host types are considered from four categories: X=(IM,IS,CUM,CUS,CIM,CIS). The first four are categories of species that are uninfected. The last two are infected versions of the *Borrellia*-competent hosts. The model assumes no host preference for the various tick stages, so larvae could feed on host type *I*, giving the following:

(A4) $$\begin{array}{cc} \dot{L_{X}} & =m_{2}L_{2}F_{X}Q_{L}X-d_{I}L_{X}-m_{3}L_{X} \end{array}$$

At this point, disease transmission may occur, giving two categories of engorged larvae followed by questing nymphs.

(A5) $$\begin{array}{cc} \dot{NU_{1}} & =m_{3}L_{IM}+m_{3}L_{IS}+m_{3}L_{CUM}+m_{3}L_{CUS} \end{array}$$

(A6) $$\begin{array}{cc} \; & \;+\left(1-p_{L}\right)\left(m_{3}L_{CIM}\right)+\left(1-p_{L}\right)\left(m_{3}L_{CIS}\right)-d_{n1}NU_{1}-m_{Ntemp}NU_{1} \end{array}$$

(A7) $$\begin{array}{cc} \dot{NI_{1}} & =p_{L}\left(m_{3}L_{CIM}\right)+p_{L}\left(m_{3}L_{CIS}\right)-d_{n1}NI_{1}-m_{Ntemp}NI_{1} \end{array}$$

(A8) $$\begin{array}{cc} \dot{NU_{2}} & =m_{Ntemp}NU_{1}-d_{n2}NU_{2}-m_{n2}NU_{2} \end{array}$$

(A9) $$\begin{array}{cc} \dot{NI_{2}} & =m_{Ntemp}NI_{1}-d_{n2}NI_{2}-m_{n2}NI_{2} \end{array}$$

Populations of infected and uninfected nymphs are tracked separately as they feed on the six host categories, as follows:

(A10) $$\begin{array}{cc} \dot{FNU_{X}} & =m_{n2}NU_{2}G_{X}Q_{X}-d_{I}FNU_{X}-m_{3}FNU_{X} \end{array}$$

(A11) $$\begin{array}{cc} \dot{FNI_{X}} & =m_{n2}NI_{2}G_{X}Q_{N}I-d_{I}FNI_{I}-m_{3}FNI_{I} \end{array}$$

Disease transmission also occurs during nymphal feeding, giving the two categories of engorged nymph and questing adult.

(A12) $$\begin{array}{cc} \; & \dot{AU_{1}}=m_{3}\left(FNU_{IM}+FNU_{IS}+FNU_{CUM}+FNU_{CUS}\right) \end{array}$$

(A13) $$\begin{array}{cc} \; & +m_{3}\left(1-p_{n}\right)\left(FNU_{CUM}+FNU_{CUS}\right)-d_{A1}AU_{1}-mfntempAU_{1} \end{array}$$

(A14) $$\begin{array}{cc} \; & \dot{AI_{1}}=m_{3}\left(FNI_{IM}+FNI_{IS}+FNI_{CUM}+FNI_{CUS}+FNI_{CIM}+FNI_{CIS}\right) \end{array}$$

(A15) $$\begin{array}{cc} \; & +m_{3}\left(p_{n}\right)\left(FNU_{CIM}+FNU_{CIS}\right)-d_{A1}AI_{1}-mfntempAI_{1} \end{array}$$

(A16) $$\begin{array}{cc} \dot{AU_{2}} & =m_{Atemp}AU_{1}-d_{A2}AU_{2}-m_{A2}\left(T\right)AI_{2} \end{array}$$

(A17) $$\begin{array}{cc} \dot{AI_{2}} & =m_{Atemp}AI_{1}-d_{A2}AI_{2}-m_{A2}\left(T\right)AI_{2} \end{array}$$

The following describe feeding adults that are uninfected and infected:

(A18) $$\begin{array}{cc} \dot{FAU_{X}} & =m_{A2}AU_{2}H_{X}Q_{X}-d_{X}FAU_{X}-m_{3}FAU_{X} \end{array}$$

(A19) $$\begin{array}{cc} \dot{FAI_{X}} & =m_{A2}AI_{2}H_{X}Q_{X}-d_{X}FAI_{X}-m_{3}FAI_{X} \end{array}$$

Fed adults come from all feeding compartments, giving the following:

(A20) $$\begin{array}{cc} \dot{A_{4}} & =m_{3}\left(\Sigma_{X}FAU_{X}+\Sigma_{X}FAI_{X}\right)-d_{A4}A_{4} \end{array}$$

The host populations and disease dynamics are as follows:

(A21) $$\begin{array}{cc} \dot{IM} & =b_{IM}I\left(1-\frac{I}{K_{I}M}\right)-d_{IM}IM \end{array}$$

(A22) $$\begin{array}{cc} \dot{IS} & =b_{IS}I\left(1-\frac{I}{K_{I}S}\right)-d_{IS}IS \end{array}$$

Disease may be transmitted by both nymphs and adults in a prevalence-dependent fashion. $J_{C}$ describes the transmission term:

(A23) $$\begin{array}{cc} \dot{CUM} & =b_{CUM}\left(CUM+CIM\right)\left(1-\frac{CUM+CIM}{K_{CM}}\right)-d_{CUM}CUM-J_{D} \end{array}$$

(A24) $$\begin{array}{cc} \dot{CIM} & =J_{D}-d_{CUM}CIM \end{array}$$

(A25) $$\begin{array}{cc} \dot{CUM} & =b_{CUS}\left(CUS+CIS\right)\left(1-\frac{CUS+CIS}{K_{CS}}\right)-d_{CIS}CUS-J_{C} \end{array}$$

(A26) $$\begin{array}{cc} \dot{CIM} & =J_{C}-d_{CUS}CIS \end{array}$$

Let $T_{i}$ be the total ticks on hosts of type *i*. Then,

(A27) $$\begin{array}{c} T_{X}=L3_{X}+FNU_{X}+FNI_{X}+FAU_{X}+FAI_{X} \end{array}$$

The transmission terms are then

(A28) $$\begin{array}{c} J_{D}=p_{CUM}CUM\left(FNI_{CUM}+FAI_{CUM}\right)./\left(T_{CUM}+\epsilon \right) \end{array}$$

(A29) $$\begin{array}{c} J_{C}=p_{CUS}CUS\left(FNI_{CUS}+FAI_{CUS}\right)/\left(T_{CUS}+\epsilon \right) \end{array}$$

For each respective host type, we have the following equations describing $Q_{x}$, the fraction of available hosts of a given type *x*, including a negligible number ϵ in the denominator for numerical stability. Let S=ΣX be the total number of hosts. As any tick may alight on any host, we have that $Q_{x}$ is the same across questing tick categories:

(A30) $$\begin{array}{cc} Q_{X} & =X/(S+\epsilon ) \end{array}$$

In this model, tick populations are bounded by the total on-host carrying capacities, $C_{X}X$, where *X* represents the populations of the three host categories I, CU, and CI, and $C_{X}$ is the per-host maximum capacity. The functional form $F_{X}$ reduces the attachment rate of a questing tick to a host as the total on-host carrying capacity for host category *X* is approached.

The functional forms F,G,H are given as the fractions of the on-host space available for further attachments and feeding for larvae, nymphs, and adults, respectively, multiplied by the probability of finding a host:

(A31) $$\begin{array}{cc} F_{X} & =max\left(\frac{q_{L}\left(C_{X}-T_{X}\right)}{C_{X}}+\epsilon ,0\right) \end{array}$$

(A32) $$\begin{array}{cc} G_{X} & =max\left(\frac{q_{N}\left(C_{X}-T_{X}\right)}{C_{X}}+\epsilon ,0\right) \end{array}$$

(A33) $$\begin{array}{cc} H_{X} & =max\left(\frac{q_{A}\left(C_{X}-T_{X}\right)}{C_{X}}+\epsilon ,0\right) \end{array}$$

The temperature (temp) and day length (LD) are given as

(A34) $$\begin{array}{cc} \; & temp=tempMean+tempVar((-10.79)cos(0.0172(t)) \end{array}$$

(A35) $$\begin{array}{cc} \; & +(-7.53)sin(0.0172(t))+(-1.212)cos(2*0.0172(t))+(-0.07472)sin(2*0.0172(t))) \end{array}$$

(A36) $$\begin{array}{c} LD=0.5+dayAmpcos(0.0172(t-174)) \end{array}$$

Maturation times are dependent on temperature and/or day length. The function Hv is the Heaviside function. For eggs, larvae, and nymphs, these are given as follows:

(A37) $$\begin{array}{c} m_{Etemp}=0.0552*exp\left(-\left(\left(temp-25.83\right)/4.946\right).^{2}\right)*Hv\left(temp-tme\right) \end{array}$$

(A38) $$\begin{array}{c} m_{Ntemp}=0.04001*exp\left(-\left(\left(temp-26.68\right)/9.533\right).^{2}\right)*Hv\left(temp-tm3\right)*\left(Hv\left(LD-LDCutnymph\right)\right) \end{array}$$

(A39) $$\begin{array}{c} m_{Atemp}=0.015865*exp\left(-\left(\left(temp-23.85\right)/9.042\right).^{2}\right)*Hv\left(temp-tmfn\right)*\left(1-Hv\left(LD-LDCutadult\right)\right) \end{array}$$

### Appendix A.2. Parameters

**Table A1:** Default parameter values are used in the numerical simulations unless otherwise mentioned. Let *X* represent the vector of hosts: X=(IM,IS,CUM,CUS,CIM,CIS).

Parameter	Value	Description
Tick parameters		
*b*	300	egg production
$d_{e}$	0.015	egg death rate
$d_{1}$	0.01	hardening larva death rate
$m_{1}$	0.033	hardening larvae maturing to questing
$d_{2}$	0.094	death rate of questing larvae
$m_{2}$	0.5	success rate of questing larvae
$d_{X}$	(0.51, 0.89, 0.73, 0.72, 0.73, 0.72)	death rate per day of feeding ticks on host *X*
$m_{3}$	0.5	drop-off rate of feeding ticks
$P_{L}$	0.2	daily probability of disease transmission from host to tick
$d_{n1}$	0.001	death rate of engorged larvae
$d_{n2}$	0.094	death rate of questing nymphs
$m_{n2}$	0.5	success rate of questing nymphs
$d_{A1}$	0.001	death rate of engorged nymphs
$d_{A2}$	0.094	death rate of questing adults
$m_{A2}$	0.5	success rate of questing adults
$d_{A4}$	0.006	death rate of engorged adults
(qL,qN,qA)	(1, 1, 1)	questing tick success rate
(tme,tm3,tmfn)	(1, 10, 15)	temperature cutoff for maturation (larvae, nymphs, adults)
ϵ	0.001	numerical stability
Host parameters		
$b_{X}$	(0.00261, 0.102, 0.00753, 0.176, n/a, n/a)	birth rate per day of uninfected host *X*,
$d_{X}$	(0.000609, 0.00129, 0.00151, 0.00345, 0.00151, 0.00345)	daily death rate of host *X*
$k_{X}$	(10, 45, 3000, 3100)	carrying capacity for (IM,IS,CUM+CIM,CUS+CIS)
$C_{X}$	(239, 176.75, 11.4, 46.84)	on host tick capacity
$p_{CUM},p_{CUS}$	(0.117, 0.6635)	rate of tick-to-host (CUM,CUS) infection
Physical parameters		
tempMean	11	mean annual temperature
tempVar	1	scaled temperature variation
dayAmp	0.0719	day length for latitude of Hanover, NH
LDcutnymph	0.569	diapause cutoffs for nymphs
LDcutadult)	0.57	diapause cutoffs for adults
Initial conditions		
$E_{0}$	6,453,100	initial number of eggs
$NU1_{0}$	856,100	initial uninfected nymphs
$NI1_{0}$	$10^{4}$	initial infected nymphs
$FNIa_{0}$	100	initial infected feeding nymphs
$AU1_{0}$	291,360	initial uninfected adults
Other ticks	0	for January 1 of run
$X_{0}$	(10, 45, 3000, 3100, 0, 0)	initial hosts of type *X*

### Appendix A.3. Data Layers and Spatial Resolution Used in MaxEnt Analysis

#### Maximum Entropy Modeling Tables

**Table A2:** Geospatial layers and data sources.

Original Data Layers	Resolution
Landsat 5 TM tiles	30 m
Landsat 7 ETM+ tiles	30 m
NLCD 2006-National	30 m (derived from Landsat 5)
NLCD 2011-National	30 m (derived from Landsat 5)
TRMM 3B42 RT tiles	0.25o (∼20 km in NH)
NAIP 2009 tiles	1 m
NAIP 2011 tiles	1 m
BioClim (WorldClim)	
Intermediate (derived layers)	
Landsat 2009 early composite	30 m
Landsat 2010 early composite	30 m
Landsat 2011 early composite	30 m
Landsat 2009 mid-composite	30 m
Landsat 2010 mid-composite	30 m
Landsat 2011 mid-composite	30 m
Landsat 2009 late composite	30 m
Landsat 2010 late composite	30 m
Landsat 2011 late composite	30 m
NLCD 2006-NH	30 m (derived from Landsat 5)
NLCD 2011-NH	31 m (derived from Landsat 5)
TRMM 2009 early composite	20 km
TRMM 2010 early composite	20 km
TRMM 2011 early composite	20 km
TRMM 2009 mid-composite	20 km
TRMM 2010 mid-composite	20 km
TRMM 2011 mid-composite	20 km
TRMM 2009 late composite	20 km
TRMM 2010 late composite	20 km
TRMM 2011 late composite	20 km
NAIP 2009 and texture derivatives	5 m
NAIP 2011 and texture derivatives	5 m

### Appendix A.4. Environmental Variables

The table below presents estimates of the relative contributions of environmental variables to the MaxEnt model. The first estimate is derived by tracking the changes in the regularized gain during each iteration of the training algorithm. Specifically, when a variable contributes to an increase in the regularized gain, its contribution value is increased, and if the absolute value of lambda decreases, the contribution is reduced. The second estimate is obtained through a permutation procedure: the values of each environmental variable are randomly permuted among the training presence and background data, and the model is then reevaluated using the permuted dataset. The resulting reduction in the training AUC is recorded and normalized to percentages. As with jackknife analyses, these variable contribution estimates should be interpreted with caution, particularly in cases where predictor variables exhibit collinearity.

**Table A3:** The contributions of specific variables in predicting the habitat of *Peromyscus leucopus*. The best predictor of habitat is the aerial imagery greenness amount.

Variable	Percent Contribution	Permutation Importance
Aerial Imagery 2011 Green Band (1 m)	22.6	38
Mean Temperature Wettest Quarter (1 km)	20.3	7
Precipitation of Coldest Quarter (1 km)	13.1	3.9
Min Temperature Coldest Month (1 km)	10.9	17
Landsat July 2009 EVI (30 m)	8.3	4.6
National Landcover Database 2011 (30 m)	7.1	7.5
Landsat May 2011 EVI (30 m)	3.1	3.1
Total Edge Low-Intensity Residential (30 m)	2.9	4.3
Precipitation Seasonality (1 km)	2.7	4.7
Core Area Evergreen Forest (30 m)	2.4	1.3
Total Edge Deciduous Forest (30 m)	1.8	3.6
GLCM Dvar on NAIP Green Band (1 m)	1.6	2.1
Core Area Deciduous Forest (30 m)	1.4	0.5
Entropy on NAIP Green Band (1 m)	0.7	1
GLCM Svar on NAIP Green Band (1 m)	0.6	0.1
GLCM Ent on NAIP Green Band (1 m)	0.5	1.1
Total Edge Evergreen Forest (30 m)	0	0.1

**Table A4:** The contributions of specific variables in predicting the habitat of *Odocoileus virginianus*. The best predictor of habitat is NLCD.

Variable	Percent Contribution	Permutation Importance
National Landcover Database 2011 (30 m)	25.9	27.7
Min Temperature Coldest Month (1 km)	23.6	14.4
Precipitation Seasonality (1 km)	16.6	8.7
Total Edge Low-Intensity Residential (30 m)	10.3	4
Aerial Imagery 2011 Green Band (1 m)	6.3	3.6
GLCM Dvar on NAIP Green Band (1 m)	6.1	2.8
Mean Temperature Wettest Quarter (1 km)	2.9	5.7
Landsat May 2011 EVI (30 m)	1.3	3.3
Precipitation of Coldest Quarter (1 km)	1.2	9.5
Core Area Evergreen Forest (30 m)	2.4	1.7
Core Area Deciduous Forest (30 m)	2.4	5.6
Total Edge Evergreen Forest (30 m)	0.8	3.7
GLCM Ent on NAIP Green Band (1 m)	0.7	1
Entropy on NAIP Green Band (1 m)	0.7	1.4
Landsat July 2009 EVI (30 m)	0.6	0.9
Total Edge Deciduous Forest (30 m)	0.5	4.2
GLCM Svar on NAIP Green Band (1 m)	0.5	1.9
